# Google Trends for health research: Its advantages, application, methodological considerations, and limitations in psychiatric and mental health infodemiology

**DOI:** 10.3389/fdata.2023.1132764

**Published:** 2023-03-27

**Authors:** Rowalt Alibudbud

**Affiliations:** Department of Sociology and Behavioral Sciences, De La Salle University, Manila, Philippines

**Keywords:** psychiatry, infodemiology, internet-based intervention, health informatics, Google Trends, methodological study, mental health, research methodology

## Abstract

The high utilization of infodemiological tools for psychiatric and mental health topics signals the emergence of a new discipline. Drawing on the definition of infodemiology by Eysenbach, this emerging field can be termed “psychiatric and mental health infodemiology,” defined as the science of distribution and determinants of information in an electronic medium, including the internet, or in a population to inform mental health services and policies. Since Google Trends is one of its popular tools, this minireview describes its advantages, application, methodological considerations, and limitations in psychiatric and mental health research. The advantage of Google Trends is the nature of its data, which may represent the actual behavior rather than their users' stated preferences in real-time through automatic anonymization. As such, it can provide readily available data about sensitive health topics like mental disorders. Therefore, Google Trends has been used to explore public concerns, interests, and behaviors about psychiatric and mental health phenomena, service providers, and specific disciplines. In this regard, several methodological can be considered by studies using Google Trends, including documenting their exact keywords, query category, time range, location, and date of retrieval. Likewise, its limitations should be accounted for in its interpretation, including restricted representation of people who use the Google search engine, limited validity in areas with low internet penetration or freedom of speech, does not provide absolute search volumes, unknown sampled queries, and limited transparency in its algorithm, especially the terms and idioms it subsumes under its “topic” keywords.

## 1. Introduction

With the ubiquity of the internet, searching and finding information has become more accessible and widespread (Bach and Wenz, 2020). Many people use the internet to understand their health and wellbeing (Bach and Wenz, 2020). Motivations for such behaviors can range from knowing more about symptoms they experience to understanding the treatment of their medical conditions (Bach and Wenz, 2020). However, increasing online health information, including misinformation, can also pose a risk to public health (Borges do Nascimento et al., 2022; World Health Organization, n.d.).

This phenomenon of “*too much information, including false or misleading information in digital platforms*,” has been termed Infodemic (World Health Organization, n.d.). Infodemics can lead to confusion and mistrust in health authorities, lengthening disease outbreaks and undermining public health responses (Borges do Nascimento et al., 2022; World Health Organization, n.d.). Thus, the World Health Organization (n.d.) recommended understanding community concerns and questions. While the rapid growth of internet information can amplify harmful messages (Borges do Nascimento et al., 2022; World Health Organization, n.d.), internet “big data” has also been used in health informatics over the past decade to understand, analyze, and predict human behavior and concerns (Mavragani and Ochoa, 2019). Eysenbach (2009) termed this field, infodemiology, a portmanteau of information and epidemiology.

Eysenbach (2009) defined infodemiology as “*the science of distribution and determinants of information in an electronic medium, specifically the Internet, or in a population, with the ultimate aim to inform public health and public policy.”* A popular tool in infodemiology is Google Trends, an open online tool that provides real-time and archived information about Google queries from 2004 onward (Nuti et al., 2014; Mavragani et al., 2018; Mavragani and Ochoa, 2019). This minireview describes its advantages, application, methodological considerations, and limitations related to psychiatric and mental health research.

## 2. Google Trends for health research

To use Google Trends for research, data are retrieved by inputting a specific keyword in its “Explore” feature (Mavragani and Ochoa, 2019). It allows the retrieval of real-time data during the last 7 days and archival data from January 2004 up to 36 h before the search is conducted by setting the desired period in various regions and countries around the globe (Mavragani and Ochoa, 2019; Google, n.d.). To display interest in a specific topic from around the world to the city level, Google Trends anonymized users' data, categorized their search queries into topics, and aggregated them together (Mavragani and Ochoa, 2019; Google, n.d.). According to Google (n.d.), “*each data point is divided by the total searches of the geography and time range it represents to compare relative popularity. Otherwise, places with the most search volume would always be ranked highest.”* The resulting numbers are then scaled from 0 to 100 “based on a topic's proportion to all searches on all topics” (Mavragani and Ochoa, 2019).

Google Trends also provides the top related queries and topics, which are the most frequently searched for terms and topics by Google users concerning the keyword of interest (Mavragani and Ochoa, 2019; Google, n.d.). Likewise, it provides rising related queries and topics, which are the terms and topics with the most significant search volume growth in the requested time and location (Mavragani and Ochoa, 2019; Google, n.d.). These queries and topics have been analyzed to understand other health concerns related to a particular keyword (Moalong et al., 2021; Alibudbud, 2022a; Alibudbud and Cleofas, 2022; Roberto et al., 2022).

### 2.1. The advantages of Google Trends for health research

Google Trends' popularity may stem from its predominant search engine market share and inherent advantages (Nuti et al., 2014; Mavragani et al., 2018; Arora et al., 2019; Mavragani and Ochoa, 2019). According to Statista (2023), Google has dominated the global search engine market since its inception and had a share of around 84.08 percent as of December 2022. This market share is far greater than other leading search engines, such as Bing (8.95%), Baidu (0.67%), and Yahoo! (2.60%). Thereby, it may represent a large proportion of the population compared to other search engine data providers. Google Trends data are also freely available, unlike other paid search engine data providers, such as Baidu Index (Fang et al., 2021). Nonetheless, it may have limited applicability in countries where Google has limited services, such as China (Fang et al., 2021; Dai and Han, 2023) (Table 1).

**Table 1:** Advantages, applications, topics, and limitations of Google Trends for health research and psychiatric and mental health infodemiology.

**Advantages of Google Trends for health research**
1. Predominant search engine market share
2. Freely available
3. Inputted queries of its users may represent the actual behavior of users rather than their preferences
4. Can provide real-time data
5. Anonymized users' information
6. Can provide readily available data about sensitive health topics (e.g., AIDS, mental disorders)
**Applications of Google Trends for health research**
1. Exploration of online public concerns, interests, and behaviors using search volumes worldwide and among and between countries
2. Predictions, nowcasting, and forecasting (limited by varying degrees of correlation)
**Psychiatric and mental health topics explored using Google Trends**
1. Mental health
2. Mental disorders (e.g., substance use disorder)
3. Mental health service providers (e.g., psychiatrists, psychologists)
4. Psychiatric sympmtoms (e.g., loneliness, self-harm)
5. Psychiatric disciplines (e.g., social psychiatry)
**Limitations of Google Trends for psychiatric and mental health infodemiology**
1. Google Trends only reflects the interests and behaviors of people with internet access and those who use the Google search engine
2. Limited validity in areas with low internet penetration or freedom of speech
3. It provides relative values rather than absolute search volumes and queries
4. The sampled queries are unknown
5. The terms and idioms under their topics keywords are unknown
6. There can be various reasons for changes in search behaviors

Another advantage is that its data represent the inputted queries of its users, thereby representing the actual behavior rather than the users' stated preferences (Mavragani and Ochoa, 2019). It can also provide real-time data that can be used to monitor behavior (Mavragani and Ochoa, 2019). Thus, it may represent difficult and time-consuming information to collect (Mavragani and Ochoa, 2019). Likewise, it provides data about web queries by automatically anonymizing the users' information (Mavragani and Ochoa, 2019; Google, n.d.). Hence, it can provide readily available data about sensitive health topics like AIDS, mental disorders, suicide, and illegal drugs (Mavragani and Ochoa, 2019). Furthermore, it has previously been used in predictions, nowcasting, and forecasting health-related topics (Mavragani and Ochoa, 2019). Hence, it has been increasingly used by health researchers over the years as an indicator of public behavior. For instance, from its inception (January 2004) to January 2014, Nuti et al. (2014) noted 70 studies that utilized Google Trends. Among them, more than a quarter (27%) were used to explore mental health topics (Nuti et al., 2014). By 2016, Mavragani et al. (2018) noted 109 studies using Google Trends.

### 2.2. Applications of Google Trends in psychiatric and mental health research

Over the years, Google Trends has been used to explore various topics and phenomena related to psychiatry and mental health (Nuti et al., 2014; Mavragani and Ochoa, 2019), such as depression (Monzani et al., 2021; Silverio-Murillo et al., 2021; de la Rosa et al., 2022; Wang et al., 2022), anxiety (Monzani et al., 2021; de la Rosa et al., 2022; Lekkas et al., 2022), suicide (Kristoufek et al., 2016; Parker et al., 2017; Silverio-Murillo et al., 2021; de la Rosa et al., 2022), insomnia (Sycińska-Dziarnowska et al., 2021; Lekkas et al., 2022), stress (Silverio-Murillo et al., 2021), substance use disorder (Parker et al., 2017; Alibudbud and Cleofas, 2022), neurocognitive disorder (Piamonte et al., 2021), and mental health, itself (Alibudbud, 2022b). It has also been used to explore online interests and concerns about mental health service providers such as psychiatrists and psychologists (Sycińska-Dziarnowska et al., 2021). Likewise, it has been utilized in psychiatric and mental health research by using search volumes worldwide and among and between countries (Monzani et al., 2021; Silverio-Murillo et al., 2021; Sycińska-Dziarnowska et al., 2021; Alibudbud, 2022b; Alibudbud and Cleofas, 2022; de la Rosa et al., 2022; Lekkas et al., 2022; Wang et al., 2022). It has also been used to analyze specific psychiatric disciplines, such as social psychiatry (Alibudbud, 2022a). It has been correlated with survey and surveillance datasets to predict, nowcast, and forecast particular psychiatric symptoms, such as loneliness and self-harm, with varying degrees of correlation (Mavragani and Ochoa, 2019; Knipe et al., 2021). Thus, future correlations with surveillance datasets of symptoms need to be explored to determine their applicability as a predictor of symptoms at a population level.

Nonetheless, Google Trends remains an excellent and popular tool for indodemiological studies that aim to explore and understand online public concerns, interests, and behaviors about health phenomena, including mental health (Nuti et al., 2014; Mavragani and Ochoa, 2019; Mavragani, 2020; Gianfredi et al., 2021; Rovetta, 2021). A review by Gianfredi et al. (2021) reiterated that studies of online information could be used to inform mental health policies and services. Thus, it can be posited that the high utilization of infodemiological tools, including Google Trends, for psychiatric and mental health topics to inform mental health services and policies can signal the emergence of a new discipline between infodemiology, psychiatric and mental health research.

Drawing on the definition of infodemiology by Eysenbach (2009), this emerging field can be termed as “psychiatric and mental health infodemiology,” and similarly, it can be defined as the *science of distribution and determinants of information in an electronic medium or in a population to inform mental health services and policies*.

### 2.3. Methodological considerations in Google Trends for psychiatric and mental health infodemiology

Google Trends remains an excellent and popular tool in psychiatric and mental health infodemiology. However, reviews suggest that studies using Google Trends may have inconsistent methodological documentation limiting the reproducibility and replicability of their findings (Nuti et al., 2014; Arora et al., 2019; Mavragani and Ochoa, 2019). Thus, this minireview describes the methodological considerations for infodemiological studies suggested by Nuti et al. (2014), Arora et al. (2019), and Mavragani and Ochoa (2019) to strengthen the replicability of results and methodological soundness of future psychiatric and mental health infodemiology. As a minimum, the three articles recommended that the following should be documented by studies utilizing Google Trends (Table 2).

**Table 2:** Suggested minimum methodological documentation and consideration by studies using Google Trends for psychiatric and mental health topics.

**Minimum methodological documentation by studies using Google Trends**
1. Keyword selection
2. Time period selection
3. Region selection
4. Query category
5. Type of search
6. Date of data retrieval

#### 2.3.1. Keyword selection

Studies should include the exact keyword used (Nuti et al., 2014; Arora et al., 2019; Mavragani and Ochoa, 2019), whether quotation marks were used (Nuti et al., 2014), any combination of keywords (i.e., whether a “+” used in combining keywords) (Arora et al., 2019; Mavragani and Ochoa, 2019), and whether the keyword was under a “mental disorder,” “topic,” or “disease” (Mavragani and Ochoa, 2019). Note that keywords under “disease” or “topics” can include keywords that fall within the category, or, as Google describes it, “*topics are a group of terms that share the same concept in any language”* (Mavragani and Ochoa, 2019). Similarly, the reason behind choosing the keyword can also be added (Nuti et al., 2014).

#### 2.3.2. Time period selection

Google Trends' search volumes and results are adjusted based on the selected location (i.e., hourly data points are shown if the period is the past 7 days, weekly data points are shown if the period is the past 5 years, and monthly data points are used if the period is beyond 5 years) (Nuti et al., 2014; Mavragani and Ochoa, 2019). Therefore, the exact period entered in Google Trends should be included.

#### 2.3.3. Region selection

Similar to time selection, search volumes and results are adjusted based on the selected location (i.e., there might be different search volume results in a country and its cities or regions) (Mavragani and Ochoa, 2019). As such, results may vary whether regions were compared (i.e., country X *vis-à-vis* country Y) (Mavragani and Ochoa, 2019). Thus, it is imperative to include the exact region or regions selected during data retrieval.

#### 2.3.4. Query category

Google Trends' users can choose from 25 topic categories and more than 300 subcategories (i.e., health as a category, mental health as a subcategory) to restrict their search (Nuti et al., 2014; Arora et al., 2019; Mavragani and Ochoa, 2019). Similarly, they can search all categories for a particular keyword. The results may vary according to the category used. For instance, if researchers are interested only in health-related searches, they should adjust the query category to “Health.”

#### 2.3.5. Type of search

Users can also conduct a search using the “Web Search,” “Image Search,” “News Search,” “Google Shopping,” and “YouTube Search” options. “Web Search” is the default option and should be selected unless researchers need to search for a particular type (Mavragani and Ochoa, 2019). In this case, it is recommended to include the rationale for these other types.

#### 2.3.6. Date of data retrieval

Google Trends search volumes may vary slightly based on the date collected (Mavragani and Ochoa, 2019). Thus, studies utilizing it should note the exact date when the data was retrieved. In cases where the infodemiological research may not be replicated due to adjustments in Google Trends' algorithm, this documentation may allow data retrieval and study replications using web-archiving services and initiatives. This is because web-archiving services and initiatives may have access to past Google Trends data (Vlassenroot et al., 2021).

### 2.4. Limitations and other considerations in using Google Trends for psychiatric and mental health infodemiology

While Google Trends has been a useful tool for psychiatric and mental health infodemiology, it has several limitations that must be accounted for. Therefore, there is a need to address some of its important limitations in its interpretation.

*First*, Google Trends only reflects the interests and behaviors of people with internet access and those who use the Google search engine (Moalong et al., 2021; Alibudbud, 2022a). Hence, it may not reflect the interests and behaviors of areas with limited internet connection and low Google market share. *Second*, a recent review by Mavragani and Ochoa (2019) has shown that it may have limited validity in areas with low internet penetration or freedom of speech. Thus, it can be recommended that traditional methods such as surveys be done to better understand public interest and concerns, especially if the study aims to understand the concerns of the whole public, including those who are not internet users. Similarly, other search engines, such as Bing, Baidu, Yandex, or Yahoo, can be explored in areas with low Google Search Engine usage (Mavragani, 2020). Additionally, Google Trends can be complemented with data from other highly utilized online platforms, such as Wikipedia, to understand further online public behaviors and concerns (Mavragani, 2020).

*Third*, Google Trends provides relative values rather than absolute search volumes and queries. Thus, a particular topic's exact number of queries for a particular topic is unknown (Nuti et al., 2014; Moalong et al., 2021). *Fourth*, Google samples its data to provide a dataset in Google Trends that represents all Google searches for a particular topic (Google, n.d.). However, the sampled queries are unknown (Mavragani and Ochoa, 2019; Rovetta, 2021). As such, it has been noted that their data may vary slightly based on the time of retrieval (Mavragani and Ochoa, 2019; Rovetta, 2021; Eichenauer et al., 2022).

*Fifth*, Google takes advantage of artificial intelligence in aggregating their search queries. However, the terms and idioms subsumed under their topic keywords are unknown due to limited transparency in Google Trends' algorithm (Nuti et al., 2014). Thus, previous studies have recommended several measures to improve reliability and replicability, such as using statistical methods (Eichenauer et al., 2022), to collect the average search volumes for several days (Rovetta, 2021), or as previously recommended, noting the setting used in their methodology including the exact keywords, query category, use of quotation marks, the rationale for keyword selection, time range, location, and date of retrieval (Nuti et al., 2014; Mavragani and Ochoa, 2019). *Sixth*, there can be various reasons for changes in search behaviors about specific topics (i.e., increased media coverage) (Bach and Wenz, 2020). Therefore, researchers must be clear on their assumptions about search behaviors and carefully interpret Google Trends data in light of their limitations.

## 3. Discussion and conclusions

Psychiatric and mental health infodemiology can be defined as the science of distribution and determinants of information in an electronic medium or in a population to inform mental health services and policies. Since Google Trends is one of its most popular tools (Nuti et al., 2014; Mavragani et al., 2018; Gianfredi et al., 2021; Sycińska-Dziarnowska et al., 2021), this minireview describes its use, advantages, methodological considerations, limitations, and application to psychiatric and mental health research over the years.

Google Trends has been applied in health research to explore public concerns, interests, and behaviors online using search volumes, as well as to predict, nowcast, and forecast. It has been used for psychiatric and mental health infodemiology to explore mental health, mental disorders, mental health service providers, psychiatric symptoms, and psychiatric disciplines. This may be because it has several advantages, including the possible representation of a large portion of the population since it has a predominant search engine market share (Nuti et al., 2014; Mavragani et al., 2018; Arora et al., 2019; Mavragani and Ochoa, 2019). In addition, its results may represent users' actual behaviors instead of their preferences (Mavragani and Ochoa, 2019). It can also provide free real-time statistics and anonymized data, including data on sensitive health topics, that can be used for predictions, nowcasting, and forecasting (Mavragani and Ochoa, 2019; Fang et al., 2021). Several methodological considerations should also be considered in using Google Trends, including documenting the keyword, time period, region, query category, type of search, and date of data retrieval. Nonetheless, it also has several limitations, including being only reflective of people with internet access and those who use the Google search engine, limited validity in regions with low internet penetration or freedom of speech, providing relative values, unknown sampled queries, and unknown terms and idioms under their topics keywords.

While generally an emerging field, its future directions may be similar to the general field of infodemiology, which is the exploration, prediction, nowcasting, and forecasting of behaviors and epidemics among and between populations (Mavragani et al., 2018; Mavragani, 2020). However, the predictive ability and correlation of infodemiological tools remain to be fully understood, developed, and adjusted to forecast future outbreaks and the prevalence of various diseases and disorders (Nuti et al., 2014; Mavragani et al., 2018; Arora et al., 2019; Knipe et al., 2021). These challenges in predictions and correlations have also been observed in psychiatric and mental health infodemiology. For instance, Google Trends has varying predictive abilities and correlations with suicidal behaviors (Kristoufek et al., 2016; Parker et al., 2017). Thus, several steps can be considered in developing psychiatric and mental health infodemiology.

First, future studies can determine and explore models and methods to improve the predictive ability and correlation of Google Trends data in predicting mental health phenomena. By doing so, it can be used to predict trends in mental health problems that need additional intervention and prevention measures. Second, since psychiatric and mental health infodemiology is a growing and emerging field, future reviews can account for the field's extent and further utilization. Lastly, infodemics may negatively impact mental health (Borges do Nascimento et al., 2022). Therefore, the applications of mental health and psychiatric infodemiology, a field at the intersection of mental health and information sciences, should be explored and developed to address infodemics and their repercussions.

## Author contributions

RA had substantial contributions to the design, drafting, revision, acquisition, interpretation, and final approval of the data and work.
